# Supplementary figures and images for: Tomato Oil Encapsulation by α-, β-, and γ-Cyclodextrins: A Comparative Study on the Formation of Supramolecular Structures, Antioxidant Activity, and Carotenoid Stability
Source: Foods. 2020 Oct 27;9(11):1553. doi: 10.3390/foods9111553 (PMC7693019; doi:10.3390/foods9111553)

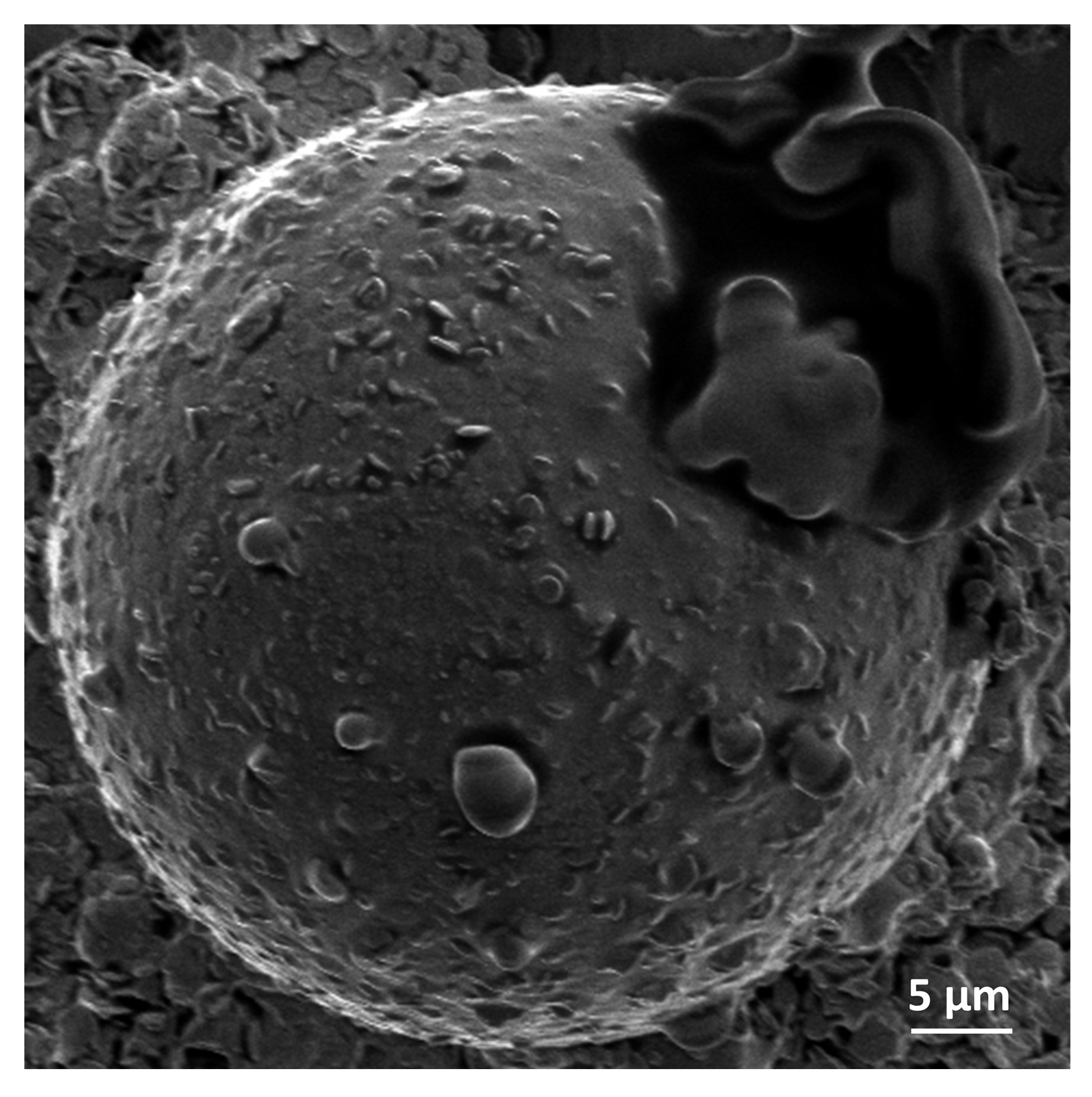

Supplement: Supplementary file 1 [file foods-09-01553-s001.zip › Supplementary figure 1.jpg]

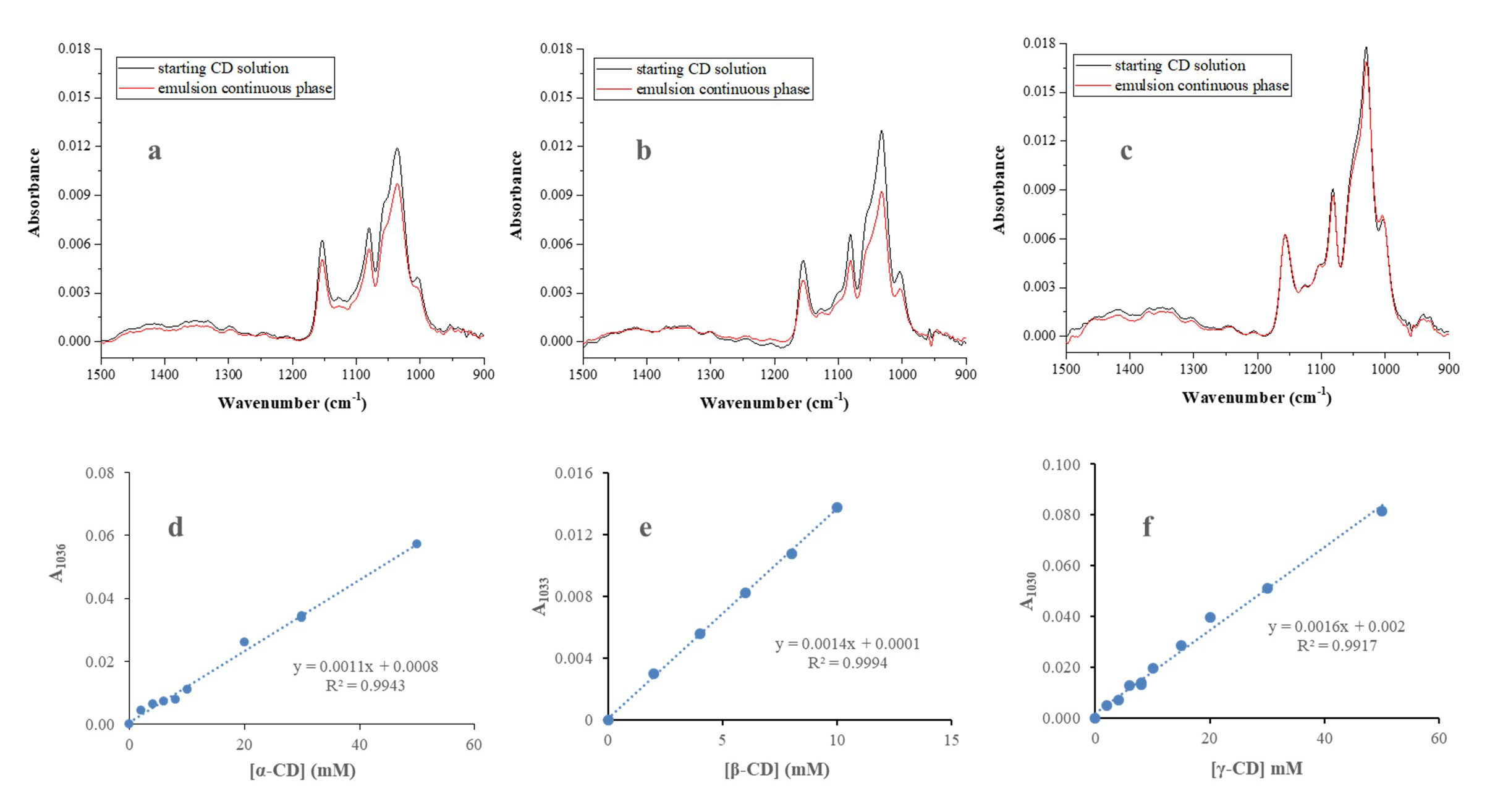

Supplement: Supplementary file 1 [file foods-09-01553-s001.zip › Supplementary figure 2.jpg]

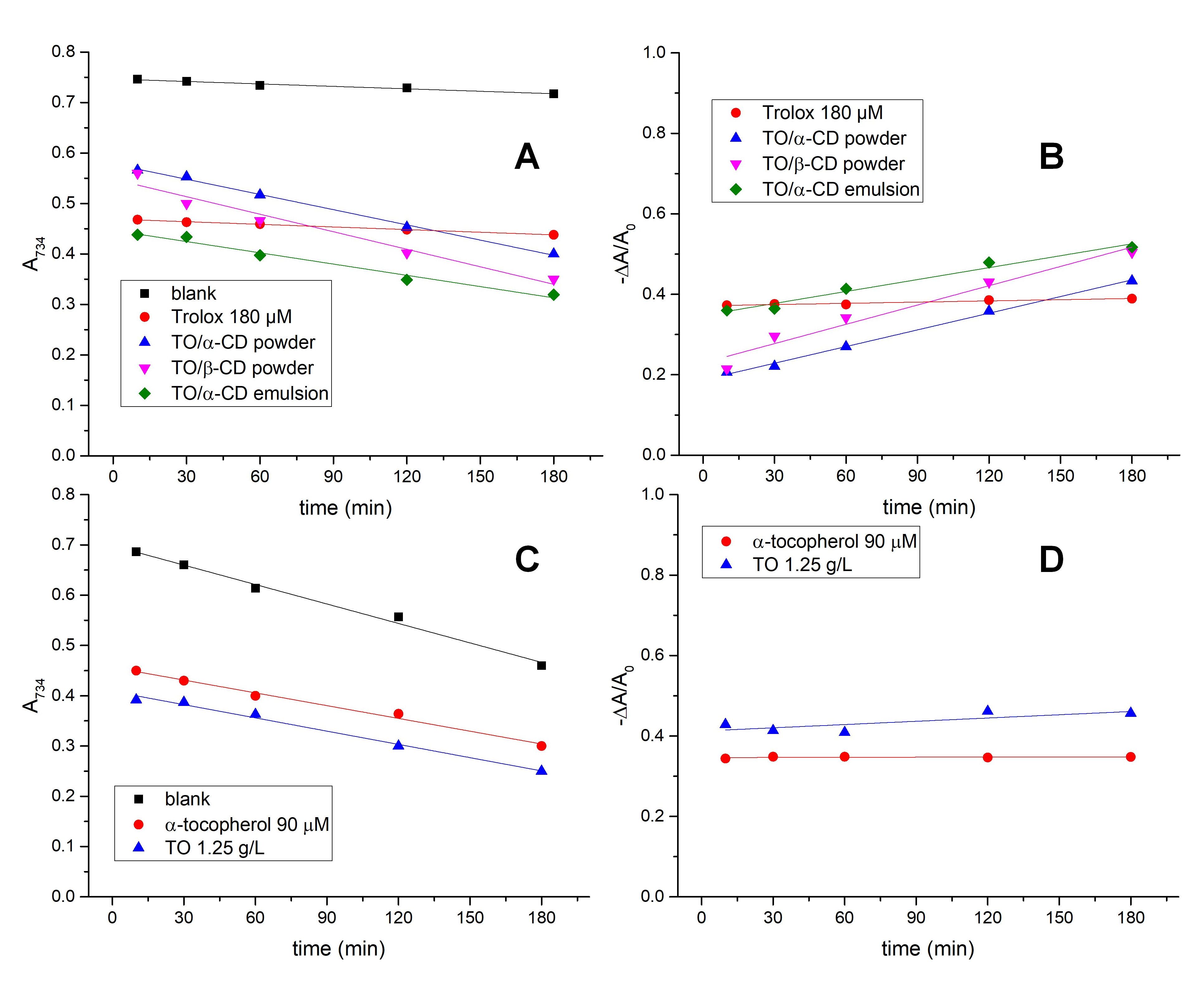

Supplement: Supplementary file 1 [file foods-09-01553-s001.zip › Supplementary figure 3.jpg]
